# Enhancing systems medicine beyond genotype data by dynamic patient signatures: having information and using it too

**DOI:** 10.3389/fgene.2013.00241

**Published:** 2013-11-19

**Authors:** Frank Emmert-Streib, Matthias Dehmer

**Affiliations:** ^1^Computational Biology and Machine Learning Laboratory, Faculty of Medicine, Health and Life Sciences, Center for Cancer Research and Cell Biology, School of Medicine, Dentistry and Biomedical Sciences, Queen's University BelfastBelfast, UK; ^2^Institute for Bioinformatics and Translational Research, UMITHall in Tyrol, Austria

**Keywords:** genome medicine, personalized medicine, next-generation sequencing data, dynOmics data, high-throughput data

## Abstract

In order to establish systems medicine, based on the results and insights from basic biological research applicable for a medical and a clinical patient care, it is essential to measure patient-based data that represent the molecular and cellular state of the patient's pathology. In this paper, we discuss potential limitations of the sole usage of static genotype data, e.g., from next-generation sequencing, for translational research. The hypothesis advocated in this paper is that dynOmics data, i.e., high-throughput data that are capable of capturing dynamic aspects of the activity of samples from patients, are important for enabling personalized medicine by complementing genotype data.

## 1. Introduction

After the completion of the human genome project (Lander et al., 2001; Venter et al., 2001; Consortium, International Human Genome Sequencing, 2004) a new era started aiming to bring results from basic biology and biomedical research into the clinic to the patients. This is often called “from bench to bedside” and defines the general idea underlying translational research and its particular realization in the form of personalized medicine. From a practical point of view, in order to accomplish such a translation of basic research results into the daily clinical routine, it is necessary to be able to generate cost-efficient patient data on the molecular and cellular level (Butte, 2008; Lussier et al., 2010). Fortunately, technological progress within the last 15 years has led to a variety of different experimental assays that provide such opportunities, even on the genomic-scale involving large portions of an organism's genes. For example, in biological research different types of “Omics” data (Ghosh and Poisson, 2009; Moreno-Risueno et al., 2010; The ENCODE Project Consortium, 2011), e.g., genomics, transcriptomics, proteomics, metabolomics and epigenomics data (Lee et al., 2002; Förster et al., 2003; Rual et al., 2005; Stelzl et al., 2005; Palsson, 2006; Sechi, 2007; Garbett et al., 2008; Yu et al., 2008) are frequently employed and could, principally, also be used in translational bioinformatics for studying patient data. Instead, currently, one could gain the feeling that genotype data from sequencing technologies, including next-generation DNA sequencing (Mardis, 2008; Shendure and Ji, 2008; Ansorge, 2009; Metzker, 2009), are dominating the discussions and the initial practical endeavours in this context (Alkan et al., 2009; Werner, 2010; Fernald et al., 2011; Zhang et al., 2011; Highnam and Mittelman, 2012; Ziegler et al., 2012). For instance, in Ng et al. (2009) direct-to-consumer (DTC) DNA tests are reviewed that are already offered by companies to identify potential disease risks of patients. Similar examples are presented in Stepanov (2010); Chin et al. (2011) with an emphasize on the utilization of DNA variations. Also, it has been argued that a genetically guided personalized medicine (GPM) has the potential to enable a patient-based treatment by utilizing sequenced DNA information from the individual patients that can be used to influence medical care decisions in the clinical practice (Welch and Kawamoto, 2012).

We would like to emphasize that it is unquestioned that genotype data, as represented for instance by single nucleotide polymorphisms (SNPs) (Collins et al., 1997; Sachidanandam et al., 2001; Wheeler et al., 2007; LaFramboise, 2009), microsatellites or whole genome sequences, provide a valuable source of information for translational bioinformatics and personalized medicine (Fernald et al., 2011). However, in this paper, we discuss potential limitations of approaches that are solely based on genotype data and emphasize the need for considering high-throughput data that are capable of capturing dynamic states and activity levels of physiological conditions of the patients. In order to distinguish such Omics high-throughput data from genotype data, we will term the latter “dynOmics” data.

This paper is organized as follows. In the next section, environmental and epigenetic influences on the genotype are discussed. Further, we characterize the static nature of genotype data. In section 3 we discuss limitation of genotype data as a consequence of the three factors discussed in section 2. In section 4 we define dynOmics data and discuss gene expression and RNA-seq data as a sources of information for such dynamic high-throughput data. Finally, in section 5 we present three application examples that utilize dynOmics data for their analysis. This paper finishes with concluding remarks.

## 2. Environmental and epigenetic factors and the static nature of genotype data

It is unquestioned that the DNA within biological cells plays an eminent role in the description of the development and evolution of an organism and the transcription regulation of the gene it encodes. Aside from the understanding of such fundamental processes, the usage of genetic information has been proven useful in studying diseases. For instance, DNA copy number variations (CNVs) (Freeman et al., 2006; Pinto et al., 2011) have been used for elucidating their role in complex disorders (McCarroll and Altshuler, 2007). Specifically, in Stephens et al. (2009); Beroukhim et al. (2010) the effect of somatic copy number alternations (SCNA) and rearrangements has been investigated for a variety of different cancer types, including breast cancer, non-small cell lung cancer, and acute lymphoblastic leukaemia. In Beroukhim et al. (2010) 158 significant regions with a focal SCNA have been identified including a large number of sites without known cancer target genes that constitute potential key players in form of tumor suppressor or oncogenes in the more than 20 different cancer types studied. Also, they found that a large majority of SCNAs can be identified in several different cancers revealing a potential similarity of the molecular pathology among these disorders. Further, in Stephens et al. (2009) it has been found that tandem duplications are particularly frequent, which might indicate a specific type of defect in DNA maintenance. A clinical connection between CNVs and patient survival was found in Kresse et al. (2010) by studying malignant fibrous histiocytoma (MFH). This finding is of particular interest because it shows a concrete example for a medical application of CNV for the diagnosis of MFH.

Despite these promising results and applications of genetic information, it is known that the information stored in the DNA alone is not sufficient to understand, and explain, the phenotypic appearance of an organism. The reason for this is that there are genotype-environment interactions that have an important influence on this as well (Falconer and Mackay, 1996; Lynch and Walsh, 1998). This means, usually, it is not possible to map a certain genotype *uniquely* to a phenotype. This genotype-environment interrelation is well know from genome-wide association studies (GWAS) and leads to a considerable increment in the complexity of the problem (Manolio et al., 2009) if one wants to apply genotype data in the medical and clinical practice, because one needs to control environmental factors. An example of environmental influences are given by mutagens. These physical or chemical agents have the ability to mutate the content of the DNA of an organism and, hence, are capable of changing the transcription of genes and the functioning of biological processes like DNA repair. Particular examples of mutagens are carcinogens, e.g., asbestos, formaldehyde, mustard gas or X-rays, that have been shown to have an influence on the development of cancer and its progression (Soffritti et al., 1989; Murthy and Testa, 1999; Hecht, 2003).

In addition to environmental influences that have an effect on the genetic information, there are epigenetic factors, e.g., DNA methylation (Ehrlich et al., 1982; Law and Jacobsen, 2010), that have also an important influence on the cell function and, hence, possibly on the phenotypic characteristics of an organism. For instance, the gene expression in normal and disease cells is known to be influenced by DNA methylation by controlling the protein-DNA binding (Richardson, 2002; Baylin, 2005; Robertson, 2005). Other examples for epigenetic factors are histone modifications and RNA interference (Egger et al., 2004; Moss and Wallrath, 2007; Ballestar and Esteller, 2008; Djupedal and Ekwall, 2009). So far it is largely unknown to what extend the epigenetic code contributes non-genetic factors to the regulation, control and maintenance of a cellular phenotype (Turner, 2007), although, within recent years important progress has been made (Dawson and Kouzarides, 2012; Sassone-Corsi et al., 2012).

A different problem in using genotype data alone for a medical application is that the DNA represents only static information about a cellular phenotype. This static information is stored in the form of nucleotide sequences representing putative programs, which may be activated under certain signaling, environmental or epigenetic conditions. That means for instance that mutations in coding or non-coding regions may or may not have an influence on the expression of genes or proteins in a particular cell type that effects the phenotype of an organism. Here, we would like to emphasize that the term “static” can also be interpreted as “passive,” because from the content of the DNA alone one cannot conclude on the activity level of its genes.

## 3. Limitations of genotype data

As a consequence of the heterogeneity induced by environmental and epigenetic factors, but also of the static nature of the DNA, there are limitations in the explanatory power of genotype data. Quantitatively, these limitations can be seen from the results of GWAS studies. Typically, GWAS studies lead only to a very small number of putative gene-associations with complex traits that are statistically significant (Sladek et al., 2007; Yeager et al., 2007). In contrast, there is usually a larger number of loci that is right below the significance threshold and, hence, these do not allow for definite conclusions. This implies that such studies suffer from a limited power and an increase is only possible by significantly increasing the number of the participating subjects (McCarthy et al., 2008). Unfortunately, this constitutes enormous practical problems for the organization and initiation of such studies and it cannot be expected that within the next few years larger studies with the required sample sizes are available which could potentially lead to the clarification of the causal involvement of genes in particular complex disorders. On a more fundamental note, we want to briefly remark that even if a locus is significantly associated with a phenotype it is not straight forward to identify the relevant gene(s) in the proximity of that locus that are implicated in the underlying disorder (Pearson and Manolio, 2008; Manolio, 2010). Further, even for significantly associated genes, their causal involvement in the explanation of a clinical phenotype is not guaranteed.

From a practical analysis perspective there is an additional problem provided by the many detectable events (variables) on the genotype-level, for instance in form of SNPs, CNVs or DNA-methylations that do not lead to actual consequences for, e.g., the survival rates of patients or other observable phenotype characteristics. This leads to a non-negligible amount of data that can be seen as *genetic noise* because it is distorting the analysis. Statistically, this constitutes non-trivial problems for the feature selection and dimension reduction of such data sets (Izenman, 2008; Clarke et al., 2009).

**Figure 1:**
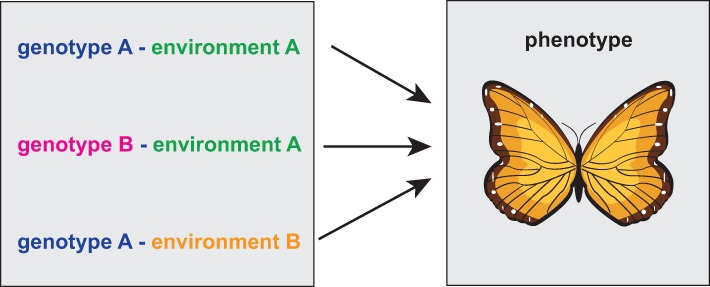
**Schematic visualization of a situation when three different genotype-environment constellations lead to the same phenotype**. Here it is important to note that the similarity of the three genotype-environment configurations can only be judged on the phenotype level of the organism.

In Figure 1 we show a visualization of the general problem. If the measurement is limited to genotype data only, it is necessary to catalog all equivalent genetic, environmental and epigenetic variations because otherwise they may be mistakenly considered as different from each other and one would expect them leading to different phenotypes.

## 4. Omics high-throughput data that provide dynamic information

In order to obtain information about the activity of molecular and cellular programs as encoded in the DNA of an organism, it is necessary to measure entities that reflect these activity states appropriately. In this respect, the expression levels of genes or proteins provide valuable information to close this gap (Speed, 2003). For example, by using DNA microarray or next-generation sequencing technologies, gene expression and RNA-seq data can be obtained representing the abundance of mRNAs in a given sample (Wang et al., 2009). By comparison with different samples, e.g., taken from a normal or a control group of patients, it is possible to infer which genes are (statistically significant) expressed or not expressed (Ge et al., 2003; Storey and Tibshirani, 2003). This information can be a valuable surrogate for the activity of these genes in their underlying physiological conditions, provided by the samples. Such a comparison is not limited to individual genes, but can also be conducted for *gene-sets* or *groups of genes* that correspond for example to biological pathways; either defined by expert knowledge or databases like Gene Ontology or KEGG (Subramanian et al., 2005; Abatangelo et al., 2009; Emmert-Streib and Glazko, 2011; Tripathi and Emmert-Streib, 2012). In this way it is possible to enable a systems approach to medicine, acknowledging the fact that genes do not operate in isolation but function collectively in a variable manner (Ahn et al., 2006; Emmert-Streib et al., 2012b).

In order to distinguish “dynamic” from “static” Omics data that allow capturing dynamic aspects of the samples from patients, we suggest the following terminology.

**DynOmics Data:** Omics data that represent dynamic aspects of a molecular and cellular system by reflecting the activity level of genes and gene products.

Particular examples for dynOmics data are transcriptomics, proteomics and metabolomics data.

**Figure 2:**
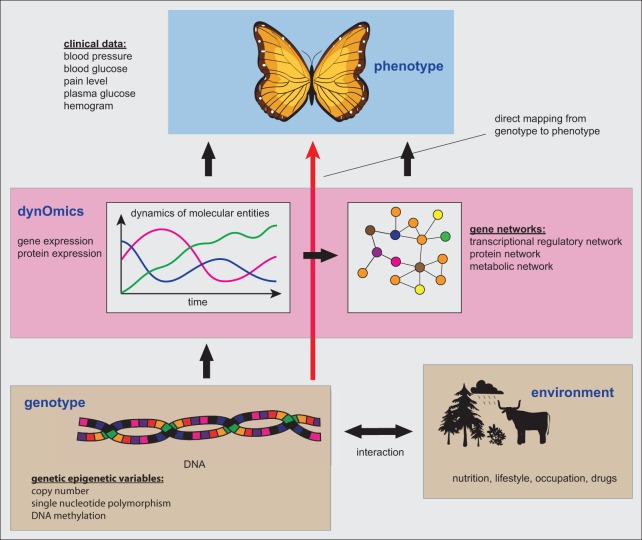
**The connection between the genetic, genomic and phenotype level**. Gene networks form one particular type of information that can be inferred from dynOmics data.

In Figure 2 we provide a summary of the connection between the genetic, genomic and phenotype level, as described in the previous sections. A direct mapping from the genotype to the phenotype, as indicated by the red arrow, could principally provide a shortcut in explaining for instance clinical patient characteristics. However, the danger is that this incurs problems by neglecting valuable information about the dynamic activity state of the cells, as represented, e.g., by the expression levels of genes or proteins. In other words, due to the static nature of genotype data this information should be seen as *potential functional information* about a patient, because information about the activity or usage of the diverse genetic programs is not captured by such data at all. Here by *potential functional information* we mean that the DNA is just a storage or a database of information (Noble, 2008) and this information is not indicative of the activity of the stored entities. For example, despite the fact that the CNV or the methylation of the DNA can change over the time of the evolution of a tumor, this does not say anything definite about the actual expression of the genes and, hence, their activation.

We would like to note that the consideration of dynamic high-throughput data, e.g., in the form of gene expression or proteomics data, does not only allow to identify differentially expressed genes or gene sets, but for sufficiently large samples sizes and variable sample conditions such data allow also to infer gene regulatory or protein networks (Belcastro et al., 2011; Emmert-Streib et al., 2012a; Emmert-Streib, 2013). These networks have the additional advantage of holding expedient clues for the molecular causes of the observed phenotypes (Emmert-Streib and Dehmer, 2011) that can be explored, e.g., by triggering follow-up experiments in the biomedical sciences. The difference to studies, e.g., utilizing DNA biomarkers to estimate the patient's disease risk (Ng et al., 2009) is that, e.g., regulatory networks provide direct insights into the molecular interaction structure of gene products (de Matos Simoes et al., 2013) and, hence, biological disease mechanisms on a level of detail that is absent in biomarker studies that are merely aiming to predict a phenotypic outcome. Furthermore, such networks can be utilized in identifying drug targets or drug mechanisms to extend traditional pharmacogenomics and pharmacodynamics approaches (Hopkins, 2008; Arrell and Terzic, 2010; Ghosh and Basu, 2012; Leung et al., 2012; Madhamshettiwar et al., 2012).

Finally, we just want to briefly mention that, strictly, there are two different types of dynOmics data that can be distinguish. The first type of dynOmics data contains *explicit information* about the temporal behavior of molecular entities as, e.g., provided by time series data of the concentration of mRNAs. In contrast, the second type of dynOmics data contains *implicit information* about the temporal behavior. An example for such dynOmics data are condition specific samples, e.g., from treatment and control patients. In the latter case, no time series (or longitudinal) data are available, yet, the data provide information about the *activity level* of genes in the form: “Is gene X active (expressed) or not.” For reasons of simplicity, we termed both types of dynamic information dynOmics data. However, as the above discussion indicates, a more refined subdivision is possible and, depending on the context, sensible.

## 5. Practical exploitation of dynomics data

Finally, we discuss a couple of particular examples of approaches where dynOmics data sets have been utilized in disease diagnoses and personalized medicine.

In Huang et al. (2010) data from the public gene expression repository gene expression omnibus (GEO), provided by the national center for biotechnology information (NCBI), have been utilized to construct a classifier for query expression profiles. Specifically, expression data from over 9000 microarray experiments have been gathered for 110 different disorders. These data sets have been used in combination with a Bayesian approach to learn a classifier for these 110 disease classes. This resulted in a method that allows to make (probabilistic) predictions about an unknown disease state as represented by a query expression profile that could be, e.g., obtained from a patient. Overall, the presented method has the capability to transform biological knowledge, as provided by the GEO database, into novel discoveries by means of the developed diagnoses tool.

For data providing information about the molecular interactions of proteins, as represented by protein interaction networks, similar approaches have been developed (Oti et al., 2006; Yang et al., 2011). For instance, in Wu et al. (2008) a method called CIPHER has been introduced that assumes that diseases with a similar phenotype are the effect of functionally related proteins that are close in a protein interaction network. In order to predict potential disease-genes based on phenotypic information about the disorder, CIPHER integrates two different types of data to define three different, connected parts. First, the online mendelian inheritance in man (OMIM) database is used to estimate the similarity between disease phenotypes by using text mining tools. Further, OMIM is also used to obtain information about gene-phenotype associations. Second, in order to assess the functional similarity between proteins the human protein interaction network is used. Here it is important to emphasize that a protein interaction network provides information about the activity of proteins in the form of their interactions and, hence, represents a type of dynOmics data.

A seminal study that demonstrates impressively the advantages of using dynOmics data has been conducted in Chen et al. (2012). This study analyzed Omics profiles, comprising genetic, transcriptomic, proteomic, metabolomic and autoantibody profiles from a single individual measured over a period of 14 months. As a result, it has been particularly highlighted that the measurement of dynamic entities is crucial if one wants to make predictions about a patient that go beyond potential effects.

## 6. Conclusions

Genome-wide high-throughput technologies provide an unprecedented opportunity for systems medicine. The major purpose of this paper has been to advocate high-throughput data that provide dynamic information about cellular states, which we termed dynOmics data. However, we would like to emphasize that this does not mean that genotype data should not be used for systems and personalized medicine. Instead, the concern of the present paper is to balance the current trend in this field that might give the misleading impression that the usage of next-generation sequencing technologies to generate, e.g., DNA-seq data is the only way to achieve the translation from basic research to medical practice. Instead, we hypothesized that dynOmics data provide an indispensable source of information representing dynamic patient signatures that should be utilized for the complementation of genotype data.

Due to the fact that gene expression data and proteomics data contain a wealth of dynamic information that is *per se* not contained in genotype data, there are inherent limitations of approaches that are solely based on such data. Furthermore, potentially, dynOmics data may represent *denoised information* compared to sequence information, because the plurality of the genetic information is decided on the functional cellular and the phenotype level. Here it is important to distinguish between “data” and “information” to comprehend the meaning of *denoised information*. Whereas “data” refer only to the measured numbers, “information” implies a semantic biological content. For this reason the fact that DNA sequencing can be performed with a higher accuracy than, e.g., the measurement of the mRNA expression, does not contradict the observation that the uncertainly in the interpretation of the functional meaning of these numbers is generally reduced from the DNA to the mRNA and the protein level.

Another important point for future developments in personalized medicine would be the *integration* of different types of genotype and dynOmics data, e.g., from transcriptomics, proteomics, metabolomics and epigenomics experiments. However, in medical practise, we are far away from such a reality (Romero et al., 2006; Ostrowski and Wyrwicz, 2009; Chan and Ginsburg, 2011) and much more basic research is necessary before we can begin translating these results into practical patient care.

### Conflict of interest statement

The authors declare that the research was conducted in the absence of any commercial or financial relationships that could be construed as a potential conflict of interest.
